# Application of immune checkpoint inhibitors in hepatocellular carcinoma: a landscape analysis of clinical trial databases

**DOI:** 10.3389/fimmu.2026.1831229

**Published:** 2026-06-10

**Authors:** Junjie Cao, Yanru Chen, Lichuan Zhang, Junyi Lou, Shuang Li, Haiquan Wen, Rong Huang, Lin Ma

**Affiliations:** 1 Department of Emergency, Affiliated Hospital of North Sichuan Medical College, Nanchong, Sichuan, China; 2 School of Clinical Medicine, North Sichuan Medical College, Nanchong, Sichuan, China; 3 Medical School of Ophthalmology & Optometry, North Sichuan Medical College, Nanchong, Sichuan, China; 4 School of Imaging Medicine, North Sichuan Medical College, Nanchong, Sichuan, China

**Keywords:** clinical trials, global development patterns, hepatocellular carcinoma, immune checkpoint inhibitors, PD-1

## Abstract

**Background:**

Hepatocellular carcinoma (HCC) is a leading global malignant tumor with poor prognosis. Immune checkpoint inhibitors (ICIs) have become a breakthrough treatment for HCC, but a systematic landscape analysis of global ICI clinical trials is lacking.

**Methods:**

Four international clinical trial databases were systematically searched up to February 14, 2026. After screening, 132 eligible trials were included and analyzed for geographic distribution, molecular targets, clinical phases, trial status and result publication rate using R 4.5.1.

**Results:**

Trials displayed a China–US dual-core pattern (95 vs. 16). PD-1/PD-L1 inhibitors were the predominant targets, while CTLA-4 inhibitor trials were scarce. Phase II was the most frequent clinical phase (59 trials). Over 80% were interventional studies; most were recruiting or of unknown status, with only 6 terminated or withdrawn. The overall result publication rate was extremely low, with 71 PD1-targeting trials and 71.4% CTLA-4 trials were unpublished.

**Conclusion:**

Global HCC ICIs trials are highly concentrated geographically and by target, with stable research progress. However, major challenges include low result translation efficiency, unbalanced target development, insufficient early/late-phase trials and uneven global collaboration. Targeted optimization is needed to promote clinical translation.

## 1 Introduction

Hepatocellular carcinoma (HCC), as the most predominant histopathological subtype of primary liver cancer (1), accounts for 75%–85% of the global incidence of liver cancer (2), and is a malignant tumor with the sixth-highest incidence and the third-highest mortality worldwide (3). Its high invasiveness, high recurrence rate, and poor prognosis in patients with advanced disease have consistently represented a focal and challenging issue in global cancer control, posing a persistent and formidable challenge to global public health systems. From the epidemiological pattern, China’s burden of HCC is particularly heavy, with the annual numbers of newly diagnosed cases and deaths accounting for nearly half of the global total, far higher than those in developed regions such as Europe and North America (3); and unlike Western countries where hepatitis C virus infection, alcoholic liver disease, and non-alcoholic fatty liver disease are the main etiologies, approximately 70%–85% of Chinese HCC patients are closely related to chronic hepatitis B virus (HBV) infection (4). This unique etiological feature means that the pathogenic mechanisms, clinical progression characteristics, and treatment response patterns of Chinese HCC show significant regional differences, and also impose special requirements for locally tailored development and application of therapeutic strategies.

Historically, the clinical management of HCC has faced numerous bottlenecks. Although early-stage patients may achieve a curative opportunity through surgical resection, liver transplantation, or local ablation, the majority are diagnosed at intermediate or advanced stages, missing the optimal window for curative therapy (5, 6). In China, the 5-year survival rate is less than 12.5% (7). For unresectable, advanced HCC, traditional treatment options are extremely limited. Previously, small-molecule targeted therapies represented by sorafenib and lenvatinib have served as the first-line standard treatment (8); however, these agents exhibit relatively low objective response rates, limited survival benefits, and are prone to primary or acquired resistance, failing to meet clinical treatment demands (9). Conventional cytotoxic chemotherapy drugs, due to significant hepatic toxicity and poor specificity, have remained limited in application for HCC, and the 5-year survival rate for patients with advanced HCC has long been below 10% (10). There remains an urgent need for therapies with novel mechanisms of action that achieve high efficacy to break this therapeutic bottleneck (11).

In recent years, the field of tumor immunotherapy has achieved leap-forward development, especially in the research and clinical application of immune checkpoint inhibitors(ICIs), which have completely disrupted the traditional treatment paradigm for solid tumors and brought hope for long-term survival to patients with advanced malignancies (12). Among them, ICIs targeting programmed death-1 (PD-1), programmed death ligand-1 (PD-L1), and cytotoxic T lymphocyte–associated antigen 4 (CTLA-4) relieve the suppressive effect of tumor cells on host immune cells, reactivating endogenous anti-tumor immune responses and achieving precise killing of tumor cells. With their durable efficacy and broad indications, they have rapidly become a breakthrough in the treatment of HCC (13–15). To date, multiple ICIs have been approved for HCC indications worldwide. Imported PD-1 inhibitors such as nivolumab and pembrolizumab were among the first approved for advanced HCC as second-line and first-line therapies, whereas the immuno-anti-angiogenic regimen of atezolizumab plus bevacizumab, owing to the significant survival benefit observed in phase III trials, has replaced traditional targeted therapies as the global first-line standard treatment for unresectable HCC (16). Meanwhile, domestically developed PD-1 inhibitors such as camrelizumab, sintilimab, and tislelizumab have also been approved for HCC indications in China. With favorable efficacy aligned to the etiological characteristics of Chinese patients, manageable safety, and greater drug accessibility, they have become core treatment options for Chinese HCC patients, further improving the global landscape of immunotherapy for HCC.

As the clinical value of ICIs in HCC continues to emerge, the global landscape of related clinical trials has grown explosively, with research scope expanding to include different targets, clinical development stages, study designs, and many countries and regions. However, existing global research is largely concentrated on the efficacy of single drugs, specific target mechanisms, or regionally limited trials, and there remains a lack of systematic, panorama-wide mapping and comprehensive analysis of global ICIs-HCC clinical trials. Key dimensions such as global geographic distribution, concentration of targets, patterns of clinical-phase deployment, trial status and progress, and differences in publication rates are still incompletely understood. Additionally, there has been no comprehensive summary and explanation of the distribution characteristics of China’s HBV-related HCC clinical trials, the global research and development (R&D) landscape, and existing development gaps, which to some extent constrain the optimization and precise deployment of global—especially Chinese—HCC immunotherapy R&D strategies.

Against this backdrop, this study systematically searched four major international clinical trial registries to comprehensively screen, extract data, and perform descriptive statistical analyses of ICIs-related clinical trials in HCC across the globe. From multiple core dimensions—geographic distribution, molecular targets and clinical phases, study types, trial status, and publication rates—we systematically dissect the current development status, distribution characteristics, and evolutionary trends of this field, identify current global R&D hotspots, core advantages, and existing problems. In particular, we focus on trials led by or involving China, and analyze issues such as imbalanced R&D activity, insufficient translation of results, and design heterogeneity across studies. This study aims to provide objective, detailed data support for the future direction of global HCC immunotherapy research, optimization of resource allocation, and exploration of combination strategies, while offering scientific reference for precision medicine practice and drug development innovation, thereby promoting sustained progress and clinical application breakthroughs in the field of HCC immunotherapy.

## 2 Method

### 2.1 Identification and screening of the study

**Figure 1:**
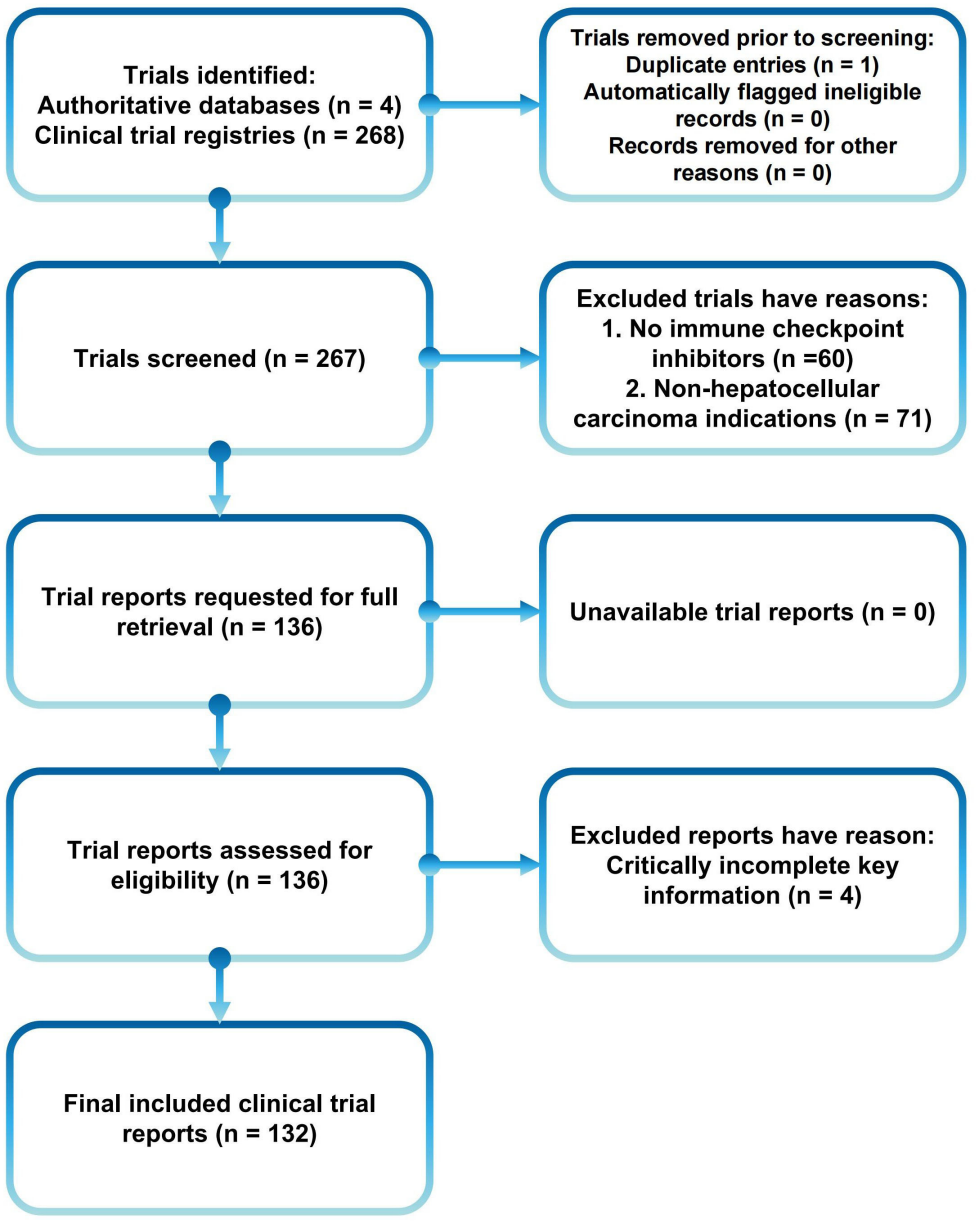
Flowchart of study selection for clinical trials of immune checkpoint inhibitors in hepatocellular carcinoma.

This study conducted a systematic analysis of clinical trials related to HCC by searching four authoritative global clinical trial registration databases (**Appendix 1**). There was no limit on the start time, and the end date was set as February 14, 2026 (search date). Using the control terms in PubMed MeSH and Embase Emtree systems, the search strategy included terms such as “Hepatocellular Carcinomas”, “Liver Cancer”, “Liver Cell Carcinomas”, “Immune Checkpoint Inhibitors”, and “Checkpoint Inhibitors, Immune”, along with their synonyms and related terms. As an example, the complete search string was: (“Carcinoma, Hepatocellular” OR “Carcinomas, Hepatocellular” OR “Hepatocellular Carcinomas” OR “Hepatocellular Carcinoma” OR Hepatoma OR Hepatomas OR “Liver Cancer, Adult” OR “Adult Liver Cancer” OR “Adult Liver Cancers” OR “Cancer, Adult Liver” OR “Cancers, Adult Liver” OR “Liver Cancers, Adult” OR “Liver Cell Carcinoma” OR “Carcinoma, Liver Cell” OR “Carcinomas, Liver Cell” OR “Cell Carcinoma, Liver” OR “Cell Carcinomas, Liver” OR “Liver Cell Carcinomas” OR “Liver Cell Carcinoma, Adult”) AND (“Immune Checkpoint Inhibitors” OR “Checkpoint Inhibitors, Immune” OR “Immune Checkpoint Blockers” OR “Checkpoint Blockers, Immune” OR “Immune Checkpoint Inhibitor” OR “Checkpoint Inhibitor, Immune” OR “CTLA-4 Inhibitors” OR “CTLA 4 Inhibitors” OR “Cytotoxic T-Lymphocyte-Associated Protein 4 Inhibitors” OR “Cytotoxic T Lymphocyte Associated Protein 4 Inhibitors” OR “Cytotoxic T-Lymphocyte-Associated Protein 4 Inhibitor” OR “Cytotoxic T Lymphocyte Associated Protein 4 Inhibitor” OR “CTLA-4 Inhibitor” OR “CTLA 4 Inhibitor” OR “PD-1 Inhibitors” OR “PD 1 Inhibitors” OR “Programmed Cell Death Protein 1 Inhibitor” OR “Programmed Cell Death Protein 1 Inhibitors” OR “PD-1 Inhibitor” OR “Inhibitor, PD-1” OR “PD 1 Inhibitor” OR “Immune Checkpoint Blockade” OR “Checkpoint Blockade, Immune” OR “Immune Checkpoint Inhibition” OR “Checkpoint Inhibition, Immune” OR “PD-L1 Inhibitors” OR “PD L1 Inhibitors” OR “Programmed Death-Ligand 1 Inhibitors” OR “Programmed Death Ligand 1 Inhibitors” OR “PD-L1 Inhibitor” OR “PD L1 Inhibitor” OR “PD-1-PD-L1 Blockade” OR “Blockade, PD-1-PD-L1” OR “PD 1 PD L1 Blockade”). The detailed search terms and records for each clinical trial database can be found in **Appendix 1**. A total of 268 potential studies were initially identified, and this screening process was independently conducted by researchers Cao and Chen (**Figure 1**), with the specific process as follows:

1. Repeated record elimination stage: By using an Excel management tool, the core information (such as trial registration number, research team, research institution, sample size, patient baseline characteristics, and research design) was compared to identify and delete duplicate records. If the core information was highly overlapping but the trial registration number was missing, the records were further determined to be duplicates through cross-validation of the study title and registration time.
2. Preliminary screening based on title, intervention, and disease type: First, perform a preliminary screening by title, intervention, and disease type to exclude clearly ineligible studies. Inclusion criteria: (i) This study focused exclusively on HCC. Participants were required to have a confirmed diagnosis of HCC via imaging examination or histopathological examination (based on biopsy specimens or surgical pathological findings), or the relevant studies should conform to the diagnostic criteria established by the American Association for the Study of Liver Diseases (AASLD) (17, 18); (ii) The core assessment content must involve immune checkpoint inhibitor–targeted therapy, either as monotherapy or in combination therapy. The search includes the following immune checkpoint inhibitors: Camrelizumab, Sintilimab, Atezolizumab, Durvalumab, Tremelimumab, Cadonilimab, Tebotelimab, etc. (whether approved or ongoing ICIs targeting CTLA-4, PD-1, PD-L1, or multi-target combinations); (iii) All trials that fully meet the above inclusion criteria will be included in the analysis, regardless of trial status (e.g., completed, recruiting, ongoing but not recruiting, unknown status), and regardless of whether results have been published. Exclusion criteria: (i) The study topic is clearly unrelated to HCC, and the study population does not meet the AASLD criteria (17, 18); (ii) The primary indication includes other cancers (e.g., non-small cell lung cancer, renal cell carcinoma) or the indication is described as solid tumor without specifying the cancer type; (iii) The study does not involve immune checkpoint inhibitor–targeted therapy or uses only non-targeted drugs; (iv) Even after reviewing the original trial registration information or contacting potential registrants to supplement information (e.g., the specific names of immune checkpoint inhibitor drugs, trial status, etc.), crucial trial details remain missing.
3. Full-text screening phase: For studies that pass the preliminary screening, obtain and review all relevant materials to further verify full eligibility. The emphasis is on confirming the exact intervention type (the type and dosing regimen of ICIs), the purity of the study population with respect to indication (whether there are subgroups of non-HCC patients), and the completeness of the study design. Studies with incomplete information or that do not meet the inclusion criteria will be excluded.

During the screening process, two investigators (Cao and Chen) independently record the number of trials included and excluded at each stage and the reasons for exclusion. If there is disagreement during screening, the dispute will be resolved by discussion to reach a consensus. If consensus cannot be reached, a third investigator (Zhang) will arbitrate, and the arbitration result will be considered the final screening conclusion.

### 2.2 Data extraction

The key data items extracted and compared included the year of study initiation and trial phase, molecular targets, trial type, trial status, biomarkers, drug name, projected sample size and follow-up duration, classification of combined pharmacotherapy, number and characteristics of HBV-related trials (including phase, target, combined medication regimen, and trial status), primary outcomes, and geographical regions where studies were conducted. Missing items in trial registries were supplemented by reviewing original registration information or contacting potential registrants. Data extraction was independently performed by two researchers (Cao and Chen). Any discrepancies or disagreements were resolved through discussion mediated by a third researcher (Zhang), who made the final decision.

### 2.3 Result release rate

This analysis aimed to evaluate the differences in the reporting rates of trial outcomes across various subgroups. Statistical approaches were adopted to assess the publication status of clinical trial results. First, official registration databases including ClinicalTrials.gov and ChiCTR were screened for labeled result postings or linked published literature. Subsequently, academic databases such as PubMed, Web of Science, Embase, Cochrane Library, CNKI, WanFang Data, and VIP were searched using trial registration numbers to retrieve matched peer-reviewed full-text articles, with key information including intervention regimens, sample sizes, and primary endpoints verified. Based on the trial completion date, trials within 12 months post-completion were preliminarily defined as pending publication. Trials exceeding 12 months post-completion with no posted results in registration databases and no matched formal journal articles, and only available as conference abstracts, preprints or internal reports, were not regarded as formal publications and were uniformly classified as unpublished clinical trial results. The analyzed subcategories were as follows: (1) Drug level: comparison of the disclosure status of trial outcomes between trials of approved established drugs and experimental drugs; (2) Target level: comparison of the disclosure status of trial outcomes among trials targeting PD-1, PD-L1, or CTLA-4; (3) Trial phase level: comparison of the disclosure status of trial outcomes across phase 0, phase I, phase I/II, phase II, phase II/III, phase III, and phase IV trials. The judgment of disclosure status was based on whether publicly accessible original or supplementary outcome data of the trial were available in the retrieved databases/registries; (4) Combination therapy regimens: single-drug regimen, dual-drug regimen, triple-drug regimen, and four-drug regimen; (5) Follow-up duration: less than 2 years, 2–5 years, and no less than 5 years; (6) Study status and primary outcome: Recruiting, Unknown, Not yet Recruiting, Completed, Active not recruiting, Terminated, and Withdrawn.

### 2.4 Statistical analysis

The statistical analysis mainly employed descriptive statistical methods. All the statistical analyses were conducted using version 4.5.1 of R and the ggplot2 package version 4.0.2 was utilized for visualization. Frequency counts were performed for categorical variables, and the results were presented in numerical (percentage) form.

## 3 Results

Cohen’s kappa coefficient was used to assess the reliability of binary inclusion/exclusion judgements on 267 trials by two independent reviewers (Cao and Chen). The observed agreement was 0.94 (251/267), with a kappa coefficient of 0.76; there were 9 trials included by Cao but excluded by Chen and 7 trials included by Chen but excluded by Cao, indicating a high level of agreement between the two reviewers. After removing duplicate records and irrelevant trials, a total of 132 trials that met the inclusion criteria were finally identified (**Appendix 2**). Temporal analysis revealed that all included trials were registered from 2017 to 2026, with the peak number occurring in 2024 (n = 27). The overall average was 13.2 trials per year(2026 trial data remains incomplete). Most trials were conducted in a single country, particularly in China, while only 4 trials were multinational (NCT05440864, NCT04696055, NCT06117891, NCT05822752). Over 80% of trials were interventional, with academic funding accounting for the majority (88.63%). Thirteen trials received mixed funding (i.e., a combination of academic and commercial support), and only 2 trials were solely commercially funded.

### 3.1 Global trial distribution

**Figure 2:**
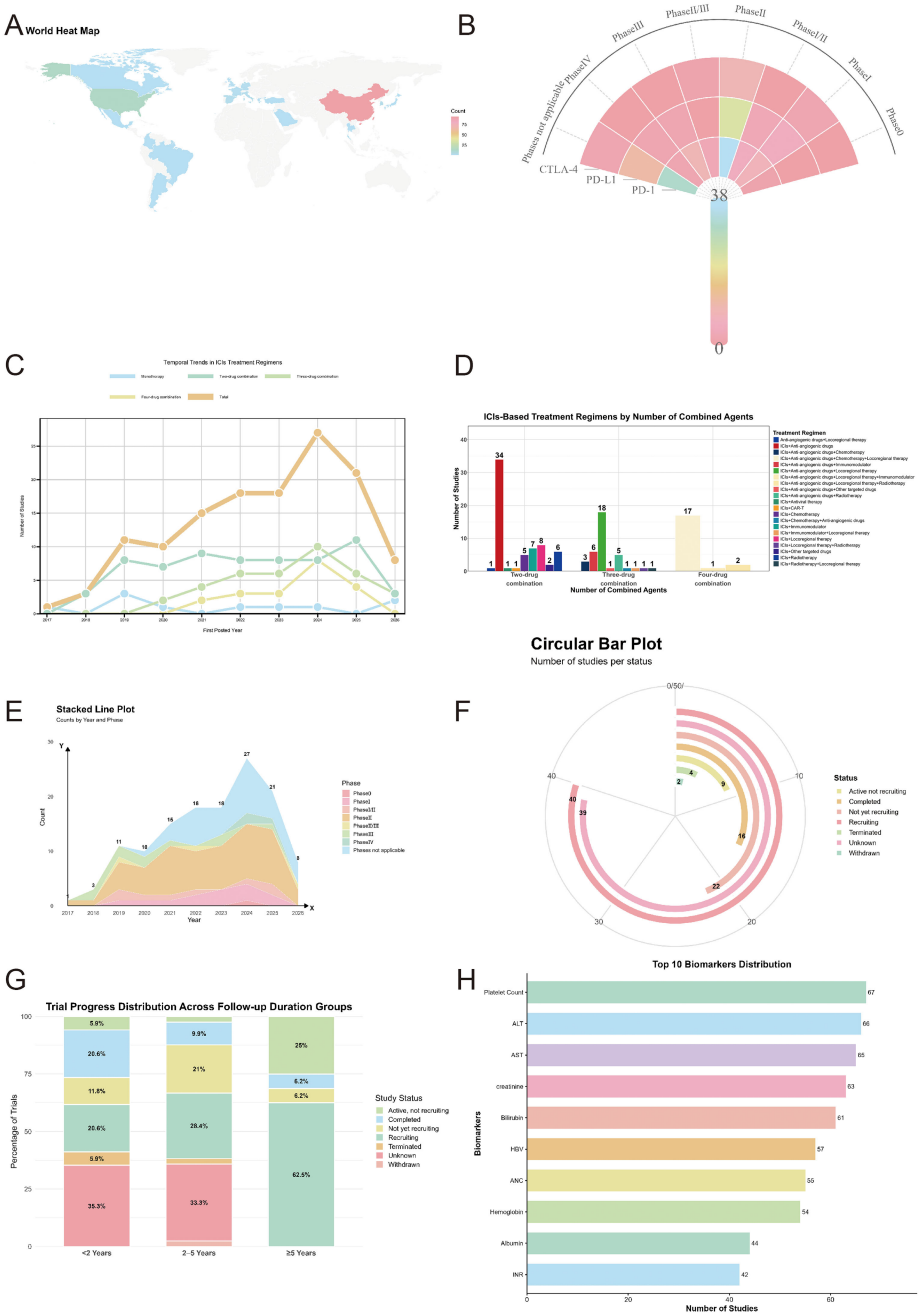
Global landscape of clinical trials of immune checkpoint inhibitors for hepatocellular carcinoma. **(A)** Geographic distribution of clinical trials worldwide; **(B)** Distribution of molecular targets and clinical phases; **(C)** Temporal trends of combination therapeutic regimens from 2017 to 2026; **(D)** Proportional composition of monotherapy, dual-agent, triple-agent, and quadruple-agent regimens; **(E)** Annual distribution and phase composition of clinical trials; **(F)** Distribution of trial states; **(G)** Distribution of the situation of the trial classified by follow-up duration; **(H)** Distribution of the core biomarkers evaluated in the clinical trial.

In terms of distribution, ICIs clinical trials present a highly concentrated and hierarchical spatial pattern (**Table 1**, **Figure 2A**). China ranks first globally with a total of 95 trials, including 92 single-center national trials, 2 trials led domestically, and 1 trials as a participating partner. It occupies an absolutely dominant position, acting as the core powerhouse for independent trial conduction while actively engaging in global clinical research collaboration.

**Table 1:** Number of trials distributed across countries.

Country	Total
China	95
United States	16
South Korea	5
Germany	2
United Kingdom	1
Italy	5
Spain	5
France	3
Japan	2
Israel	1
Canada	1
Argentina	1
Brazil	1
Colombia	1
Greece	1
Mexico	1
Saudi Arabia	1
Thailand	1
Turkey	1
Poland	1

The United States ranks second with 16 trials in total, comprising 12 single-country trials, 3 leading trials, and 1 participating trials. Notably, it has the largest number of participating trials worldwide and functions as a pivotal hub for global multicenter clinical collaboration. South Korea, Germany, the United Kingdom and other European and Asian countries only conduct occasional independent single-country trials.

Specifically, South Korea joins 5 collaborative trials and Germany leads 1 trial, with their overall involvement remaining relatively limited. By contrast, Italy, Spain, France and Japan have no independent single-country trials and mainly participate in global multicenter research as collaborating partners. Spain is involved in 5 trials; Italy and South Korea each participate in 5 trials; France takes part in 3 trials and Japan in 3 trials, with France also demonstrating partial capacity to lead collaborative research. Countries including Israel, Canada, Argentina, Brazil, Colombia, Greece, Mexico, Saudi Arabia, Thailand, Turkey and Poland each participate in merely one collaborative trial, belonging to marginal participating nations in this field. Overall, the global distribution forms a dual-core landscape led by China as the absolute core and supported by the United States. Independent clinical trials are highly concentrated in these two nations, whereas multicenter collaborations extend to a wider range of countries, forming a distinct distribution feature of core-country leadership supplemented by multi-participant international collaboration.

### 3.2 Target mechanisms and phase characteristics

In the joint distribution of molecular targets and clinical phases, ICIs clinical trials exhibit a highly centralized and distinctive pattern (**Figure 2B**). From the target perspective, PD-1 and PD-L1 inhibitors constitute the absolute research core, whereas CTLA-4 inhibitors account for a markedly lower proportion: PD-1 inhibitors are broadly deployed across all clinical stages, covering Phase 0 through Phase IV and “Phases not applicable,” representing the most active target category. PD-L1 inhibitors are largely concentrated in Phase I, Phase II, and combination phases, also showing prominence in mid-stage clinical research. CTLA-4 inhibitors primarily participate in Phase II and combination-phase trials, and the overall scale and coverage are much smaller than those of PD-1/PD-L1 inhibitors. From the clinical phase perspective, Phase II is the most central trial phase, occupying the largest share across all three major targets and serving as the core vehicle for efficacy validation and regimen optimization. There are also many trials in the “Phases not applicable” category, which predominantly consist of observational or real-world studies and are especially prominent among PD-1/PD-L1 inhibitors. Early phases (Phase I, Phase I/II) and late phases (Phase III, Phase IV) are relatively few and largely focused on PD-1 inhibitors, reflecting that early exploration and late confirmation remain PD-1 pathway–driven. Additionally, multi-target combination trials (e.g., PD-1 with PD-L1, CTLA-4 with PD-L1) exist in small numbers and are mainly in mid-stage clinical phases, indicating the field’s gradual expansion toward combination targeting and multi-pathway synergy. Overall, the field’s clinical trials center on PD-1/PD-L1 as the core targets, with Phase II as the core stage, while also accommodating multi-target combinations and non-phase-specific research; this yields a distribution feature of “highly concentrated targets, mid-stage predominant”.

### 3.3 Analysis of combination therapeutic regimens

Statistical analyses were performed on the number and category of combined regimens related to ICIs (**Figures 2C, D**). Temporal distribution by year revealed that ICI treatment was predominantly administered in combination regimens, with an overall annual upward trend in combination intensity, and the period from 2024 to 2025 represented the peak application of combination strategies. Stratified by the number of combined agents, monotherapy regimens (single-agent use) were mainly observed between 2017 and 2020, with a marked decline in proportion after 2021 and only a small number remaining in use until 2026. Dual-agent regimens constituted the most commonly used foundational strategy throughout the entire study period, primarily consisting of ICIs combined with anti-angiogenic agents, regional therapy, chemotherapy, radiotherapy, or immunomodulators, and maintained high-frequency application from 2022 to 2025. Triple-agent regimens increased rapidly starting in 2021 and became the dominant combination modality during 2023 to 2025, with the most prevalent patterns being ICIs plus anti-angiogenic agents plus regional therapy, as well as ICIs plus anti-angiogenic agents plus chemotherapy/radiotherapy/immunomodulators. Quadruple and higher-order combination regimens increased remarkably after 2022 and accounted for the highest proportion in 2024–2025, typically represented by ICIs combined with anti-angiogenic agents, chemotherapy and regional therapy, or ICIs combined with anti-angiogenic agents and regional therapy plus radiotherapy or immunomodulators, indicating the widespread adoption of complex combination strategies. In summary, ICI treatment has gradually evolved from early monotherapy dominance into a stepped combination pattern centered on dual-agent regimens as the foundation, triple-agent regimens as the mainstay, and quadruple-agent regimens as intensified strategies, with both the complexity and application frequency of combination therapy increasing year by year.

### 3.4 Phase and temporal characteristics of trials

From the combined distribution of time and clinical phase, ICI clinical trials exhibit clear annual trends and phase differentiation (**Figure 2E**). The total number of trials started with 1 in 2017; from 2019 to 2023, an apparent steady growth phase occurred; in 2024, the number peaked at 27; in 2025 it declined to 21; in 2026 it further declined to 8, showing an overall inverted-V pattern and indicating that 2024 was the most active year for trial activity in this field. In terms of phase composition, Phases not applicable trials dominate across all years, and their fluctuations closely track the overall trend; these non-interventional studies are the primary driver of year-to-year increases and decreases in trial counts, reflecting the central role of non-interventional research in temporal dynamics. In traditional phased trials, Phase II trials are the most numerous and maintain relatively active progress, aligning with the core role of mid-stage efficacy validation. Phase I, Phase III, and Phase IV trials are fewer in number and exhibit relatively stable fluctuations; early exploratory phases (Phase 0, Phase I/II, Phase II/III) are very limited, nearly negligible. Overall, annual trial counts are driven primarily by non-phase-specific studies; traditional phased trials display a more stable cadence, with Phase II remaining the core focus for traditional phased clinical trials.

### 3.5 Status of immune checkpoint inhibitors clinical trials

The status distribution of clinical trials related to ICIs for HCC exhibits significant disparities across follow-up durations (**Figures 2F, G**). Overall, trials are predominantly in the recruiting (40 trials) and unknown status (39 trials) categories, with comparable numbers between the two groups. These are followed by not yet recruiting (22 trials), completed (16 trials), and active but not recruiting (9 trials). The numbers of terminated (4 trials) and withdrawn (2 trials) trials are relatively small, resulting in an extremely low overall rate of trial termination and withdrawal, which indicates stable progress of research in this field.

Stratified analysis revealed that among trials with a follow-up duration of less than 2 years, unknown status (35.3%) and recruiting status (20.6%) accounted for the largest proportions, followed by completed (20.6%) and not yet recruiting (11.8%). For trials with a follow-up duration of 2 to 5 years, unknown status (33.3%) and recruiting status (28.4%) were the predominant categories, while completed (9.9%) and active but not recruiting (9.9%) trials showed relatively balanced proportions. In trials with a follow-up duration of 5 years or longer, recruiting status accounted for as high as 62.5%, followed by active but not recruiting status (25%), whereas both completed and not yet recruiting statuses constituted only 6.2% each, with no trials categorized as unknown, terminated, or withdrawn. This distribution pattern suggests that trials with long follow-up cycles are mainly characterized by ongoing participant enrollment and implementation, whereas trials with short and medium follow-up durations present a more dispersed status distribution. The high proportion of trials with unknown status in short-term follow-up cohorts is largely attributable to incomplete progress disclosure due to limited follow-up time. Further analysis of withdrawal causes demonstrated that the two withdrawn trials were unrelated to safety or efficacy concerns; instead, they stemmed from adjustments in the sponsor’s research and development strategy and insufficient early participant enrollment, rather than inherent deficiencies in the clinical value of the investigational agents.

### 3.6 Distribution of core biomarkers in clinical trials of immune checkpoint inhibitors for hepatocellular carcinoma

Among the clinical trials of immune checkpoint inhibitors included in this study for hepatocellular carcinoma, the top 10 most frequently evaluated biomarkers were all routine clinical detection indicators, reflecting the high research attention paid to patients' baseline liver function, hematological status and virological characteristics in this field (**Figure 2H**). Platelet Count was investigated in the largest number of trials (67 trials), followed by alanine transaminase (ALT, 66 trials), aspartate transaminase (AST, 65 trials), Creatinine (63 trials), Bilirubin (61 trials), hepatitis B virus (HBV, 57 trials), absolute neutrophil count (ANC, 55 trials), Hemoglobin (54 trials), Albumin (44 trials), and international normalized ratio (INR, 42 trials). Overall, liver function indicators (ALT, AST, Bilirubin, Albumin), renal function indicator (Creatinine), hematological indicators (Platelet Count, ANC, Hemoglobin, INR), and HBV infection status constitute the most concerned baseline evaluation system in current immunotherapy research, serving as the most commonly used stratification and monitoring tools in clinical trials of immunotherapy for hepatocellular carcinoma.

### 3.7 Analysis of characteristics associated with HBV status

**Figure 3:**
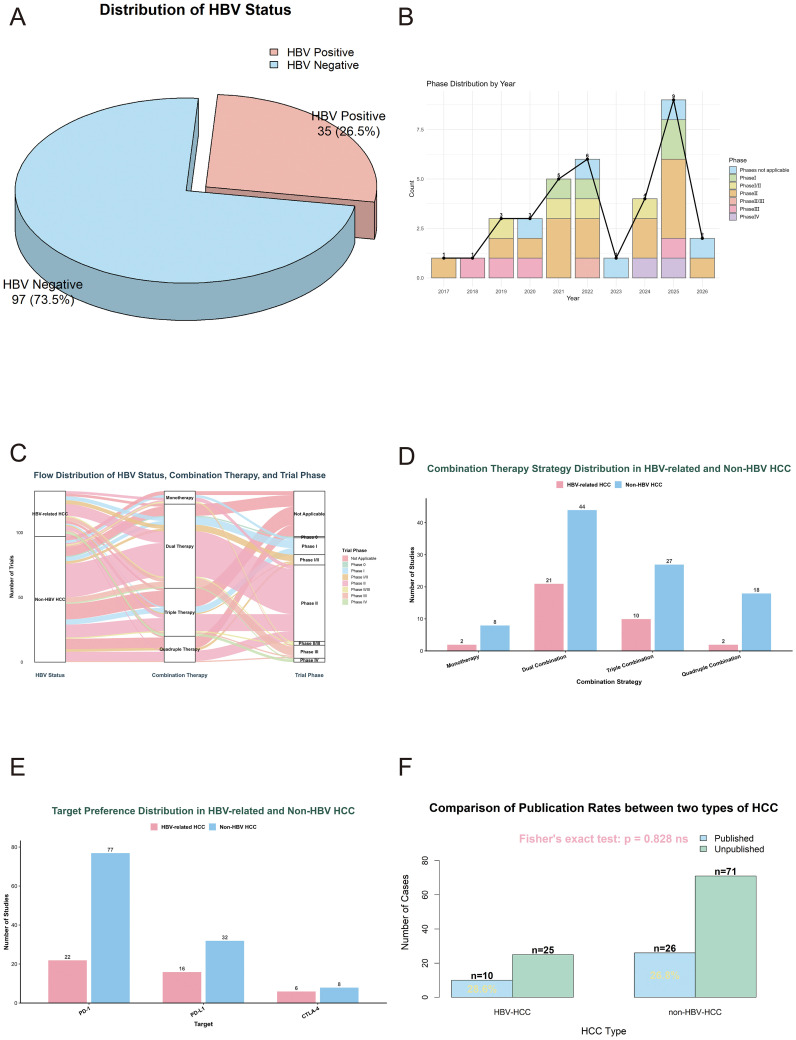
Characteristics of HBV-related and non-HBV-related clinical trials. **(A)** Proportion of HBV-positive and HBV-negative trials; **(B)** Temporal phase trends of HBV-related HCC trials; **(C)** Phase distribution of HBV-related and non-HBV-related trials; **(D)** Comparison of therapeutic regimens between HBV-related and non-HBV-related trials; **(E)** Target preference in HBV-related and non-HBV-related trials; **(F)** Publication rate comparison between HBV-related and non-HBV-related trials.

Among the clinical trials of immune checkpoint inhibitors included in this study for HCC, HBV-negative studies accounted for a higher proportion (97 trials, 73.5%), while HBV-positive studies numbered 35 trials (26.5%) (**Figure 3A**). Temporal trend analysis of trial phases for HBV-related HCC trials revealed that exploratory Phase I/II studies predominated in the early period (**Figure 3B**). Stratified analysis of treatment regimens showed that the number of monotherapy studies was lower for HBV-related HCC (2 trials) than for non-HBV-related HCC (8 trials). Dual combination therapy constituted the largest number of studies for both HBV-related HCC (21 trials) and non-HBV-related HCC (44 trials). In triple and quadruple combination therapy settings, non-HBV-related HCC still had more studies than HBV-related HCC (**Figure 3D**). Flow analysis further demonstrated that dual combination regimens dominated both categories of trials, with Phase II trials serving as the core developmental stage and no obvious discrepancy in phase distribution between the two groups (**Figure 3C**). Analysis of target preference indicated that PD-1 inhibitors were the predominant choice in both trial categories. Studies involving PD-1 inhibitors were markedly more numerous in non-HBV-related HCC (77 trials) than in HBV-related HCC (22 trials). Studies investigating PD-L1 and CTLA-4 inhibitors also exhibited a higher proportional distribution in non-HBV-related HCC (**Figure 3E**). Comparison of publication rates showed a publication rate of 28.6% (10/35) for HBV-related HCC trials and 26.8% (26/97) for non-HBV-related HCC trials, with no statistically significant difference (Fisher’s exact test, p = 0.828) (**Figure 3F**).

### 3.8 Multidimensional characteristic analysis of the publication rate of clinical trials of immune checkpoint inhibitors in hepatocellular carcinoma

**Figure 4:**
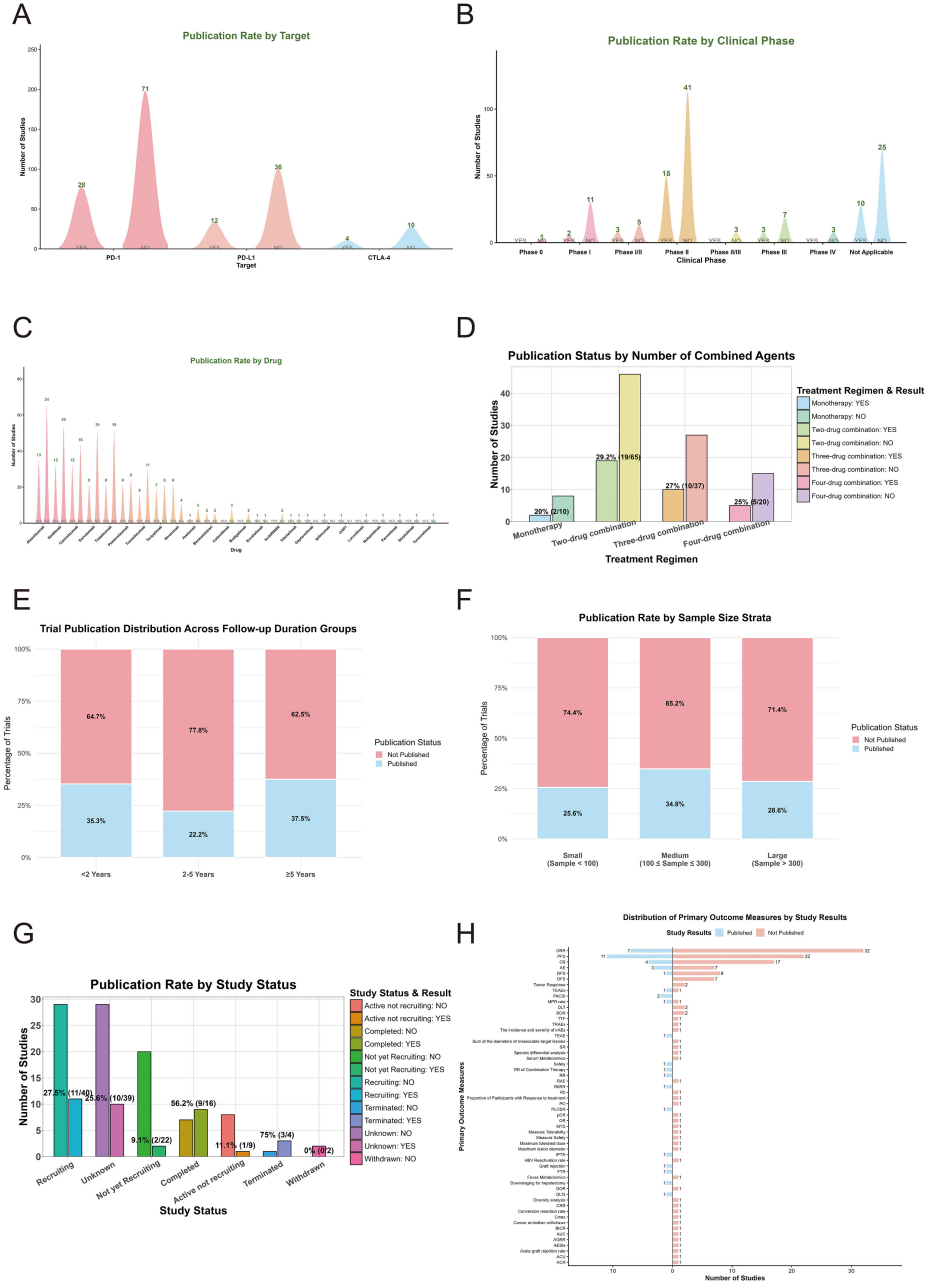
Multidimensional analysis of publication rates in clinical trials. **(A)** Publication rate by target type (PD-1, PD-L1, CTLA-4); **(B)** Publication rate by clinical phase; **(C)** Publication rate by individual immune checkpoint inhibitor drug; **(D)** Publication rate by combination therapeutic regimen; **(E)** Publication rate by follow-up duration; **(F)** Publication rate by sample size; **(G)** Publication rate by trial status; **(H)** Distribution of primary endpoints in published and unpublished trials.

This study analyzed the distribution characteristics of the publication rate of included clinical trials of ICIs for HCC from multiple dimensions, including target type, clinical stage, combination regimen, follow-up duration, sample size, and trial status (**Figure 4**). At the target level, trials related to PD-1 inhibitors were the most numerous (99 trials), followed by PD-L1 inhibitors (48 trials) and CTLA-4 inhibitors (14 trials). The distribution trend of publication rates was highly consistent with that of trial numbers, with PD-1 inhibitor-related trials exhibiting the highest number of publication (**Figure 4A**). Stratified by clinical stage, phase II trials were significantly more abundant than those of other stages (59 trials), followed by trials with “not applicable” staging (35 trials), phase I trials (13 trials), and phase III trials (10 trials). Completed phase II trials had the highest number of publications, while phase I and phase III trials showed relatively lower number of publications, indicating that mid-term confirmatory trials tend to achieve academic publication preferentially (**Figure 4B**). Among immune checkpoint inhibitors, Atezolizumab (13 published trials), Sintilimab (12 published trials), Camrelizumab (12 published trials), Durvalumab (8 published trials), Tislelizumab (8 published trials), and Pembrolizumab (8 published trials) ranked at the forefront in terms of both trial quantity and publication rate. In contrast, other PD-1/PD-L1 inhibitors, CTLA-4 inhibitors, and investigational drugs generally had lower trial numbers and publication rates, suggesting that marketed mainstream PD-1/PD-L1 inhibitors serve as the core agents for current immunotherapy research and achievement transformation in hepatocellular carcinoma (**Figure 4C**). Stratified by combination regimen, dual-drug combination regimens accounted for the largest number of trials (65 trials), followed by triple-drug regimens (37 trials), quadruple-drug regimens (20 trials), and monotherapy regimens (2 trials). Among published trials, dual-drug combination regimens occupied the highest proportion, whereas quadruple-drug regimens showed a slightly lower publication rate (**Figure 4D**). Stratified by follow-up duration, trials with a follow-up duration of 2–5 years had the lowest publication rate (22.2%), while those with follow-up duration of less than 2 years (35.3%) and no less than 5 years (37.5%) had relatively higher publication rates. This implies that trials with medium-term follow-up may not yet complete publication due to unfinished data collation, whereas trials with short follow-up achieve early maturation of endpoint events and those with long follow-up adopt more mature study designs, resulting in higher publication rates (**Figure 4E**). Stratified by sample size, trials with a moderate sample size (100–300 cases) had a publication rate of 34.8%, slightly higher than that of small-sample trials (<100 cases, 25.6%) and large-sample trials (>300 cases, 28.6%) (**Figure 4F**). Stratified by trial status, completed trials achieved a publication rate of 56.2%, which was markedly higher than that of trials in recruitment (27.5%), not yet recruiting (9.1%), terminated trials (75% with only 4 included trials), and withdrawn trials (0%). Trials with unknown status had a publication rate of 25.6%, clearly demonstrating that completed trials constitute the primary source of published research findings (**Figure 4G**). The distribution of primary endpoint indicators revealed that studies taking overall survival (OS), progression-free survival (PFS), and objective response rate (ORR) as primary endpoints accounted for the highest proportion among published trials, with no significant difference in the distribution of endpoint indicators compared with unpublished trials (**Figure 4H**).

## 4 Discussion

This study performed a panoramic analysis of global clinical trials of immune checkpoint inhibitors for hepatocellular carcinoma from 2017 to 2026. The results revealed a highly concentrated geographical distribution of trials, with China dominating with 95 trials and the United States serving as an important contributor with 16 trials; multinational collaborations collectively formed a research and development landscape led by core countries. The included studies exhibited good reliability following consistency testing, and a total of 132 trials were ultimately enrolled. The number of trials reached its peak in 2024, which were predominantly single-country and interventional studies, with academic grants constituting the overwhelming majority of funding sources. Trial targets and disease stages were highly concentrated, with PD-1/PD-L1 as the core targets and phase II trials as the pivotal stage for efficacy validation and regimen optimization. Combination regimens were predominant, forming a stepwise combination model based on dual-agent regimens, dominated by triple-agent regimens, and intensified by quadruple-agent regimens, with the peak application occurring during 2024–2025. Most trials were in the recruiting or unknown status, while the rates of trial termination and withdrawal were extremely low and unrelated to safety and efficacy outcomes. Core biomarkers mainly included liver function, hematological parameters, renal function indicators, and HBV-related markers, collectively establishing the baseline evaluation system. HBV-negative studies accounted for a larger proportion, whereas HBV-related and non-HBV-related studies showed consistent trends in trial stage, therapeutic regimen, and target distribution, with no significant difference in publication rates. Publication rates were jointly influenced by target type, trial phase, combination regimen, follow-up duration, sample size, and trial status. Higher publication rates were observed in trials targeting PD-1 inhibitors, phase II studies, dual-agent regimens, trials with moderate sample sizes, and completed trials. Overall survival, progression-free survival, and objective response rate served as the primary endpoints in published studies.

Currently, multiple PD-1/PD-L1 inhibitors have been approved for clinical treatment of HCC, becoming one of the core treatment modalities for intermediate- to advanced-stage HCC (19, 20). For example, PD-1 inhibitors such as nivolumab and pembrolizumab have successively been approved for second-line and first-line treatment of HCC, and the atezolizumab plus bevacizumab regimen has ushered in a new era of immunotherapy combined with anti-angiogenic therapy for HCC, becoming a first-line standard treatment for unresectable HCC (21, 22). Domestic PD-1 inhibitors in China, such as sintilimab and camrelizumab, have also been approved for HCC indications, and with favorable efficacy and accessibility, have become important treatment options for Chinese HCC patients (23, 24). Additionally, the dual immunotherapy regimen of the CLTA-4 inhibitor Tremelimumab in combination with Nivolumab has also been approved for HCC treatment, providing a new therapeutic direction for patients who fail immunotherapy with monotherapy (25). There are clear differences in indications and dosing regimens among different ICIs; some PD-1 inhibitors are approved as second-line monotherapy, whereas combination regimens are more often approved as first-line therapy, and some domestically produced drugs show more targeted efficacy in HBV-related HCC patients, aligning with the epidemiological characteristics of HCC in China.

Multidimensional analyses in this study reveal that global clinical trials of ICIs-HCC exhibit a highly concentrated dual-core pattern centered on China and the United States. China ranks first worldwide with 95 trials, followed by the United States with 16 trials, and the two countries together account for nearly 95.45% of all trials. Most other countries participate mainly through collaborative projects, forming an unbalanced distribution characterized by dominance of core countries and marginal participation of other nations. While this pattern accelerates research and development progress, it substantially limits the generalizability of research findings worldwide. Hepatocellular carcinoma in China is predominantly attributed to HBV infection, whereas HCV infection, alcoholic liver disease, and non-alcoholic fatty liver disease constitute the major etiologies in Europe and North America (4). Such etiological differences directly lead to population heterogeneity in immunotherapeutic efficacy, safety profiles, and biomarker expression. The current trial design overwhelmingly dominated by China and the United States fails to cover the population characteristics and clinical practice patterns in high-burden regions including Africa, Southeast Asia, and South America, resulting in insufficient external validity of research outcomes. Furthermore, the proportion of global multinational collaborative trials remains extremely low. Trials conducted solely within a single country predominate in China, and international multicenter trials led by the United States are limited. Inadequate data sharing and cross-regional validation further weaken the global applicability of clinical evidence. Analysis of target and phase distribution indicates that PD-1/PD-L1 inhibitors hold an absolutely dominant position, while research on CTLA-4 inhibitors remains scarce. Trials are highly concentrated in phase II, with inadequate phase I exploratory studies and phase III confirmatory trials. The existing target homogeneity and developmental phase discontinuity restrict the discovery of novel targets and the efficiency of clinical translation. Combination therapy has formed a stepwise developmental trend with dual-agent regimens as the foundation, triple-agent regimens as the mainstream, and quadruple-agent regimens as intensive strategies. Nevertheless, complex therapeutic regimens increase trial difficulty and prolong research cycles, which serve as key contributors to the relatively low publication rate of relevant findings. Data on trial status and publication demonstrate that ongoing recruiting and unknown-status trials account for over 60% of the total, with a low proportion of completed trials and an overall extremely low publication rate. This phenomenon is prone to inducing publication bias, leading to overrepresentation of positive results and redundant waste of research resources. Biomarker analyses primarily focus on routine liver and renal function as well as hematological and HBV-related indicators, while exploration of precise immune predictive biomarkers such as PD-L1, TMB, and MSI remains insufficient, hindering the advancement of personalized therapy. Subgroup analysis of HBV-related trials shows that such trials account for merely 26.5% of the total, a scale mismatched with the substantial disease burden in China, which impedes the establishment of evidence-based diagnosis and treatment strategies tailored to the Chinese population.

A total of 132 clinical trials of ICIs-HCC were included in this study. The current research landscape was systematically summarized from multiple dimensions including target distribution, combination therapeutic strategies, and clinical developmental stages. This analysis highlights promising research directions in this field and indicates that the immunotherapy system for hepatocellular carcinoma will continue to be optimized, with the potential to further improve long-term survival outcomes of patients. Currently, clinical trials combining ICIs with anti-angiogenic agents, chemotherapy, and locoregional therapies have been widely conducted, which effectively overcome the efficacy limitations of monotherapy and further enhance the clinical benefits of immunotherapy. Meanwhile, China has accumulated abundant dedicated research on HBV-related hepatocellular carcinoma, which closely aligns with the disease characteristics and clinical diagnostic and therapeutic needs of the Chinese population, providing robust evidence-based support for the formulation of localized and individualized treatment regimens (26, 27). In addition, observational studies enable the rapid accumulation of real-world clinical data, offering critical evidence for rational clinical medication, efficacy evaluation, and safety management.

The publication rate of clinical trials serves as a crucial indicator for measuring research maturity, translational efficiency, and academic output quality in a given field (28). Multidimensional analyses in this study reveal significant disparities in publication rates across different targets, clinical phases, combination regimens, follow-up durations, sample sizes, and trial statuses. Such discrepancies are not primarily attributed to differences in the selection of outcome endpoints, but rather arise from the combined effects of research design complexity, implementation cycle, data maturation timeline, and the acceptance preferences of academic journals. Studies on mainstream marketed PD-1/PD-L1 inhibitors tend to yield more published findings, while publications related to CTLA-4 inhibitors remain limited, reflecting a prevailing tendency among clinical researchers to focus on established agents with solid evidence bases and clear clinical application prospects. Phase II trials exhibit prominent publication advantages over exploratory phase I and confirmatory phase III trials, owing to their dual value in efficacy exploration and regimen optimization, moderate research duration, and timely maturation of short-term endpoints. By contrast, complex multi-agent combination regimens involve intricate design, numerous confounding factors, and higher outcome uncertainty, making it more challenging to generate definitive publishable conclusions compared with conventional dual-agent regimens, thereby objectively widening the gap in publication rates. Follow-up duration and sample size also profoundly influence the pace of academic dissemination. Trials with interim follow-up often experience publication delays due to ongoing data collation, whereas studies with short follow-up can rapidly reach endpoint events and those with long follow-up possess well-established design frameworks, both facilitating timely academic output. Studies with moderate sample sizes strike an optimal balance between statistical power and clinical feasibility, exhibiting superior translational potential for publication relative to small-sample studies with insufficient persuasiveness and large-sample trials that entail substantial time and resource costs. Trial progress status represents a decisive factor affecting publication rates; only successfully completed trials possess comprehensive datasets suitable for publication, while numerous trials in recruitment, pending initiation, or undefined status remain stagnated for extended periods, directly reducing overall academic productivity.

The imbalanced publication pattern also implies substantial underlying challenges. Insufficient long-term publication of certain trial types may induce publication bias, whereby negative and neutral findings remain underreported while positive results are over disseminated. This may overestimate the genuine efficacy of immunotherapy, hinder clinicians’ objective assessment of benefits and risks, and undermine the comprehensiveness of the evidence-based medical system (29, 30). Furthermore, the large number of undisclosed and ambiguously defined trials leads to opaque research information, triggers redundant project approval of homogeneous studies, wastes research funding and participant resources (31), and impedes the systematic accumulation of data from characteristic subgroups such as HBV-related hepatocellular carcinoma, thereby delaying the update of domestic clinical guidelines and the refinement of individualized treatment strategies. Notably, the proportions of trial termination and withdrawal in this cohort are exceedingly low and unrelated to drug efficacy or safety, being merely driven by corporate research layout adjustments and enrollment difficulties. This demonstrates that the overall research and development landscape in this field remains stable, without systematic safety concerns or a shrinking pipeline of clinical investigation.

Future research should advance the optimization of ICIs in HCC treatment from multiple dimensions to tackle existing bottlenecks in research development and clinical implementation. Notably, the field presents a relatively high non-publication rate: as many as 71 PD-1 targeted trials remain unpublished, and 28.6% of CTLA-4 inhibitors have been formally published, leaving substantial clinical data untapped and underutilized. Hence, it is urgent to optimize the dissemination mechanism of clinical trial outcomes, facilitate the full disclosure of unpublished data particularly negative and neutral results, avoid redundant research, and improve overall research and development efficiency (32). Meanwhile, the target development distribution is unbalanced, with CTLA-4 inhibitor research remaining small in scale and mainly concentrated on combination regimens; future studies should strengthen the optimization of CTLA-4 monotherapy and the exploration of novel combination strategies, and develop innovative immune checkpoint targets to diversify therapeutic alternatives.

Drug resistance remains the core clinical challenge of HCC immunotherapy, and current investigations on immune resistance reversal strategies and predictive biomarkers are still insufficient (33). In-depth exploration of the HCC immune microenvironment is warranted, and specific biomarkers including PD-L1 expression, tumor mutational burden (TMB), and microsatellite instability (MSI) should be further validated to realize precise patient stratification and improve the targeting accuracy and efficacy of immunotherapy (34), future preclinical and translational research should integrate standardized animal models of muscle atrophy (35)to systematically evaluate immune checkpoint inhibitor-associated muscle toxicity and explore potential mitigation strategies. In addition, both early- and late-phase trials are limited in scale. Future research should prioritize Phase II studies of mainstream targets and dual-combination regimens with definite clinical value and concise, mature trial design via rational planning of sample size and follow-up duration, strengthen dose exploration and safety evaluation in Phase I trials, accelerate confirmatory Phase III trials to promote the clinical approval of promising regimens, and attach importance to Phase IV post-marketing surveillance to clarify long-term efficacy and safety profiles. Moreover, emphasis should be placed on the result disclosure of ongoing, negative and neutral studies, as well as standardized release of trial progress information, so as to reduce academic lag and publication bias caused by numerous trials with unknown status. This strategy can not only optimize the allocation of scientific research resources, but also enrich evidence-based medical evidence, driving the standardized, efficient and rigorous development of clinical research on HCC immunotherapy.

At the same time, targeted optimization is needed to address the global imbalance in research networks and the relatively low proportion of interventional studies. On one hand, strengthening international multicenter collaboration between China, the United States, and other high-incidence countries of HCC, integrating global research resources, and conducting large-scale, multicenter clinical trials will help to improve the global applicability of study results. On the other hand, a balance between observational research and interventional research should be sought: while conducting high-quality randomized controlled trials to support clinical guidelines based on higher levels of evidence, observational studies should also be utilized to collect real-world data. In addition, to address enrollment failures in clinical trials, trial design should be optimized so that inclusion criteria better reflect real-world clinical practice, to improve patient enrollment efficiency and reduce trial termination due to nonclinical factors.

This study has several limitations. First, restricted by the accessibility of publicly registered data, the depth of subgroup analysis was insufficient. Fine stratification based on key factors such as etiology, ethnicity, age, and liver function was not performed, which limits the applicability of the conclusions to specific populations. Second, only descriptive statistical analyses were adopted in this study. Owing to the extremely low disclosure rate of trial outcomes, systematic extraction and evaluation of efficacy and safety endpoints could not be implemented, and relevant evidence needs to be supplemented by subsequent systematic reviews. In addition, this study did not incorporate a unified quality evaluation framework for clinical trials, nor did it conduct stratified analyses regarding global geographical heterogeneity. These shortcomings partially compromise the completeness, reliability, and regional generalizability of the research findings.

In summary, ICIs have become a breakthrough therapy for HCC, bringing new survival hope to patients with intermediate- to advanced-stage disease. Globally, clinical trials have formed a mature development pattern with China and the United States as dual cores, with PD-1/PD-L1 as the central targets, and phase II as the core stage; the overall development process is stable and outlook is optimistic. However, clinical application and development still face several challenges, such as low translational efficiency, uneven distribution of targets, unresolved resistance, and insufficient global collaboration. These issues require solutions through improved data-disclosure mechanisms, deeper investigation of targets and biomarkers, optimization of combination strategies, and strengthened international multicenter collaboration. As precision medicine and tumor immunology advance, ICIs are expected to further combine with chemotherapy, anti-angiogenic therapy, locoregional therapy, and targeted therapy to form more efficient and precise comprehensive treatment regimens, becoming an important component of HCC multimodal therapy and continually enhancing clinical outcomes for patients with liver cancer.

## 5 Conclusion

This study systematically analyzed 132 global clinical trials of ICIs for HCC up to February 2026. The global research landscape presents a China-US dual-core pattern, with China conducting 95 trials and the United States 16 trials. Interventional studies account for the majority (106/132), PD-1/PD-L1 inhibitors are the core targets, while CTLA-4 inhibitor research is extremely limited. Phase II trials serve as the main clinical stage, and combination therapy has become the mainstream strategy, showing a stepped development trend of dual-drug as the basis, triple-drug as the main body, and quadruple-drug as the enhancement. Trial numbers peaked in 2024, with a low overall termination and withdrawal rate and stable research progress. Nevertheless, prominent issues remain, including an extremely low result publication rate, unbalanced target research layout, insufficient early and late-phase trials, and uneven global cooperation. HBV-related HCC trials account for a relatively small proportion, with no significant difference in publication rate compared with non-HBV-related trials. In the future, it is necessary to improve the trial result disclosure mechanism, strengthen the research of CTLA-4 and new targets, optimize the clinical stage layout, and enhance international multi-center collaboration, so as to promote the clinical transformation of ICIs therapy and further improve the prognosis of HCC patients.

## Data Availability

The original contributions presented in the study are included in the article/**Supplementary Material**. Further inquiries can be directed to the corresponding author.

## References

[1] Liu Y Ouyang L Mao C Chen Y Li T Liu N . PCDHB14 promotes ferroptosis and is a novel tumor suppressor in hepatocellular carcinoma. Oncogene. (2022) 41:3570–83. doi: 10.1038/s41388-022-02370-2 35688944

[2] Chen Q Chen AZ Jia G Li J Zheng C Chen K . Molecular imaging of tumor microenvironment to assess the effects of locoregional treatment for hepatocellular carcinoma. Hepatol Commun. (2022) 6:652–64. doi: 10.1002/hep4.1850 34738743 PMC8948593

[3] Sung H Ferlay J Siegel RL Laversanne M Soerjomataram I Jemal A . Global cancer statistics 2020: GLOBOCAN estimates of incidence and mortality worldwide for 36 cancers in 185 countries. CA Cancer J Clin. (2021) 71:209–49. doi: 10.3322/caac.21660 33538338

[4] Yang JD Hainaut P Gores GJ Amadou A Plymoth A Roberts LR . A global view of hepatocellular carcinoma: trends, risk, prevention and management. Nat Rev Gastroenterol Hepatol. (2019) 16:589–604. doi: 10.1038/s41575-019-0186-y 31439937 PMC6813818

[5] Li J Liang Y Chen X Ou Z Wang Q Luo W . Prognostic value of lymphatic vessel density in the capsule of early-stage hepatocellular carcinoma: implications for postoperative recurrence risk. Front Immunol. (2026) 17:1714314. doi: 10.3389/fimmu.2026.1714314 41685307 PMC12891066

[6] Vogel A Meyer T Sapisochin G Salem R Saborowski A . Hepatocellular carcinoma. Lancet. (2022) 400:1345–62. doi: 10.1016/S0140-6736(22)01200-4 36084663

[7] Wang W Wei C . Advances in the early diagnosis of hepatocellular carcinoma. Genes Dis. (2020) 7:308–19. doi: 10.1016/j.gendis.2020.01.014 32884985 PMC7452544

[8] Llovet JM Ricci S Mazzaferro V Hilgard P Gane E Blanc J . Sorafenib in advanced hepatocellular carcinoma. N Engl J Med. (2008) 359:378–90. doi: 10.1056/NEJMoa0708857 18650514

[9] Jin H Shi Y Lv Y Yuan S Ramirez CFA Lieftink C . EGFR activation limits the response of liver cancer to lenvatinib. Nature. (2021) 595:730–34. doi: 10.1038/s41586-021-03741-7 34290403

[10] Abou-Alfa GK Lau G Kudo M Chan SL Kelley RK Furuse J . Tremelimumab plus durvalumab in unresectable hepatocellular carcinoma. Nejm Evid. (2022) 1:EVIDoa2100070. doi: 10.1056/EVIDoa2100070 38319892

[11] Mak L Liu K Chirapongsathorn S Yew KC Tamaki N Rajaram RB . Liver diseases and hepatocellular carcinoma in the Asia-Pacific region: burden, trends, challenges and future directions. Nat Rev Gastroenterol Hepatol. (2024) 21:834–51. doi: 10.1038/s41575-024-00967-4 39147893

[12] Yau T Galle PR Decaens T Sangro B Qin S Da Fonseca LG . Nivolumab plus ipilimumab versus lenvatinib or sorafenib as first-line treatment for unresectable hepatocellular carcinoma (CheckMate 9DW): an open-label, randomised, phase 3 trial. Lancet. (2025) 405:1851–64. doi: 10.1016/S0140-6736(25)00403-9 40349714

[13] Tong J Tan Y Ouyang W Chang H . Targeting immune checkpoints in hepatocellular carcinoma therapy: toward combination strategies with curative potential. Exp Hematol Oncol. (2025) 14:65. doi: 10.1186/s40164-025-00636-5 40317077 PMC12046748

[14] Harding JJ . Immune checkpoint blockade in advanced hepatocellular carcinoma: an update and critical review of ongoing clinical trials. Future Oncol. (2018) 14:2293–302. doi: 10.2217/fon-2018-0008 29663837 PMC7444624

[15] El-Khoueiry AB Sangro B Yau T Crocenzi TS Kudo M Hsu C . Nivolumab in patients with advanced hepatocellular carcinoma (CheckMate 040): an open-label, non-comparative, phase 1/2 dose escalation and expansion trial. Lancet. (2017) 389:2492–502. doi: 10.1016/S0140-6736(17)31046-2 28434648 PMC7539326

[16] Gao X Zhao R Ma H Zuo S . Efficacy and safety of atezolizumab plus bevacizumab treatment for advanced hepatocellular carcinoma in the real world: a single-arm meta-analysis. BMC Cancer. (2023) 23:635. doi: 10.1186/s12885-023-11112-w 37415136 PMC10327339

[17] Taddei TH Brown DB Yarchoan M Mendiratta-Lala M Llovet JM . Critical update: AASLD practice guidance on prevention, diagnosis, and treatment of hepatocellular carcinoma. Hepatology. (2025) 82:272–74. doi: 10.1097/HEP.0000000000001269 39992051

[18] Singal AG Llovet JM Yarchoan M Mehta N Heimbach JK Dawson LA . AASLD practice guidance on prevention, diagnosis, and treatment of hepatocellular carcinoma. Hepatology. (2023) 78:1922–65. doi: 10.1097/HEP.0000000000000466 37199193 PMC10663390

[19] Nan YM Miao TG . Advances in targeted and immune therapies for hepatocellular carcinoma. Zhonghua Gan Zang Bing Za Zhi. (2022) 30:905–11. doi: 10.3760/cma.j.cn501113-20220624-00344 36299181 PMC12770260

[20] Li Q Han J Yang Y Chen Y . PD-1/PD-L1 checkpoint inhibitors in advanced hepatocellular carcinoma immunotherapy. Front Immunol. (2022) 13:1070961. doi: 10.3389/fimmu.2022.1070961 36601120 PMC9806143

[21] Liu Z Liu X Liang J Liu Y Hou X Zhang M . Immunotherapy for hepatocellular carcinoma: current status and future prospects. Front Immunol. (2021) 12:765101. doi: 10.3389/fimmu.2021.765101 34675942 PMC8524467

[22] Kudo M Finn RS Galle PR Zhu AX Ducreux M Cheng A . IMbrave150: Efficacy and safety of atezolizumab plus bevacizumab versus sorafenib in patients with Barcelona Clinic Liver Cancer stage B unresectable hepatocellular carcinoma: an exploratory analysis of the phase III study. Liver Cancer. (2023) 12:238–50. doi: 10.1159/000528272 37767068 PMC10521324

[23] Qin S Chan SL Gu S Bai Y Ren Z Lin X . Camrelizumab plus rivoceranib versus sorafenib as first-line therapy for unresectable hepatocellular carcinoma (CARES-310): a randomised, open-label, international phase 3 study. Lancet. (2023) 402:1133–46. doi: 10.1016/S0140-6736(23)00961-3 37499670

[24] Zeng X Jia Y Chen H Luo Q Zhao H Liang G . A real-world analysis of survival and cost-effectiveness of sintilimab plus bevacizumab biosimilar regimen in patients with advanced hepatocellular carcinoma. J Cancer Res Clin Oncol. (2023) 149:9213–19. doi: 10.1007/s00432-023-04775-2 37188985 PMC10185451

[25] Sangro B Chan SL Kelley RK Lau G Kudo M Sukeepaisarnjaroen W . Four-year overall survival update from the phase III HIMALAYA study of tremelimumab plus durvalumab in unresectable hepatocellular carcinoma. Ann Oncol. (2024) 35:448–57. doi: 10.1016/j.annonc.2024.02.005 38382875

[26] Chen W Zheng R Baade PD Zhang S Zeng H Bray F . Cancer statistics in China, 2015. CA Cancer J Clin. (2016) 66:115–32. doi: 10.3322/caac.21338 26808342

[27] Zheng R Qu C Zhang S Zeng H Sun K Gu X . Liver cancer incidence and mortality in China: temporal trends and projections to 2030. Chin J Cancer Res. (2018) 30:571–79. doi: 10.21147/j.issn.1000-9604.2018.06.01 30700925 PMC6328503

[28] Crockett LK Okoli GN Neilson CJ Rabbani R Abou-Setta AM Klassen TP . Publication of randomized clinical trials in pediatric research: a follow-up study. JAMA Netw Open. (2018) 1:e180156. doi: 10.1001/jamanetworkopen.2018.0156 30646048 PMC6324306

[29] Song F Parekh S Hooper L Loke YK Ryder J Sutton AJ . Dissemination and publication of research findings: an updated review of related biases. Health Technol Assess. (2010) 14:1–193. doi: 10.3310/hta14080 20181324

[30] Carlisle BG Gönen M Fernandez L Del Paggio JC Kimmelman J . Drug approvals in crowded drug classes potentially reflect efficacy overestimates and false positivity: a portfolio analysis of immune checkpoint inhibitor trials. J Clin Epidemiol. (2026) 193:112182. doi: 10.1016/j.jclinepi.2026.112182 41692173

[31] Ferland-Beckham C Petty S Prager E Harmon N Haas M Jeromin A . Leveling the playing field: a new initiative to publish negative and replication data in brain trauma. Neurotrauma Rep. (2020) 1:146–47. doi: 10.1089/neur.2020.0055 34223538 PMC8240893

[32] Chan A Tetzlaff JM Altman DG Laupacis A Gøtzsche PC Krleža-Jerić K . SPIRIT 2013 statement: defining standard protocol items for clinical trials. Ann Intern Med. (2013) 158:200–07. doi: 10.7326/0003-4819-158-3-201302050-00583 23295957 PMC5114123

[33] He M Liu Y Chen S Deng H Feng C Qiao S . Serum amyloid A promotes glycolysis of neutrophils during PD-1 blockade resistance in hepatocellular carcinoma. Nat Commun. (2024) 15:1754. doi: 10.1038/s41467-024-46118-w 38409200 PMC10897330

[34] Zhang N Yang X Piao M Xun Z Wang Y Ning C . Biomarkers and prognostic factors of PD-1/PD-L1 inhibitor-based therapy in patients with advanced hepatocellular carcinoma. biomark Res. (2024) 12:26. doi: 10.1186/s40364-023-00535-z 38355603 PMC10865587

[35] Zhang G Hu F Huang T Ma X Cheng Y Liu X . The recent development, application, and future prospects of muscle atrophy animal models. MedComm - Future Med. (2024) 3:e70008. doi: 10.1002/mef2.70008 41531421

