# Supplementary material for: Application of immune checkpoint inhibitors in hepatocellular carcinoma: a landscape analysis of clinical trial databases
Source: Front Immunol. 2026 Jun 10;17:1831229. doi: 10.3389/fimmu.2026.1831229 (PMC13290964; doi:10.3389/fimmu.2026.1831229)
Supplement: Supplementary file 2 [file SupplementaryFile2.docx]

| Appendix 2 Clinical Trials Included in the Study | | | | | |
| --- | --- | --- | --- | --- | --- |
| Trail Number | Study Status | Study Results | Phases | StudyType | First Posted |
| NCT06991907 | Completed | YES | Phases not applicable | Interventional | 2025 |
| NCT04310709 | Completed | YES | PhaseⅡ | Interventional | 2020 |
| NCT05307926 | Completed | NO | Phases not applicable | Observational | 2022 |
| NCT07184424 | Completed | NO | Phases not applicable | Interventional | 2025 |
| NCT05453383 | Unknown | NO | PhaseⅡ | Interventional | 2022 |
| NCT06375317 | Not yet Recruiting | NO | PhaseⅡ | Interventional | 2024 |
| NCT06708650 | Not yet Recruiting | NO | Phases not applicable | Interventional | 2024 |
| NCT06978933 | Recruiting | NO | PhaseⅡ | Interventional | 2025 |
| NCT07157969 | Recruiting | YES | PhaseⅡ | Interventional | 2025 |
| NCT04680598 | Unknown | NO | Phases not applicable | Observational | 2020 |
| NCT05332821 | Unknown | YES | Phases not applicable | Observational | 2022 |
| NCT07410715 | Not yet Recruiting | NO | Phases not applicable | Observational | 2026 |
| NCT05332496 | Unknown | YES | Phases not applicable | Observational | 2022 |
| NCT06867432 | Not yet Recruiting | NO | PhaseⅡ | Interventional | 2025 |
| NCT07379489 | Not yet Recruiting | NO | Phases not applicable | Observational | 2026 |
| NCT07230314 | Not yet Recruiting | NO | PhaseⅡ | Interventional | 2025 |
| NCT04220944 | Active not recruiting | NO | PhaseⅠ | Interventional | 2020 |
| NCT06631326 | Completed | YES | Phases not applicable | Observational | 2024 |
| NCT05162898 | Unknown | NO | Phases not applicable | Interventional | 2021 |
| NCT06248554 | Recruiting | NO | Phases not applicable | Interventional | 2024 |
| NCT05440864 | Recruiting | YES | PhaseⅡ | Interventional | 2022 |
| NCT05048017 | Unknown | YES | PhaseⅡ | Interventional | 2021 |
| NCT05910970 | Unknown | YES | PhaseⅢ | Interventional | 2023 |
| NCT05717738 | Recruiting | YES | Phases not applicable | Observational | 2023 |
| NCT03970616 | Terminated | YES | PhaseⅠ/Ⅱ | Interventional | 2019 |
| NCT07100405 | Recruiting | NO | Phases not applicable | Observational | 2025 |
| NCT03937830 | Active not recruiting | NO | PhaseⅡ | Interventional | 2019 |
| NCT04229355 | Unknown | YES | PhaseⅢ | Interventional | 2020 |
| NCT03949231 | Recruiting | NO | PhaseⅢ | Interventional | 2019 |
| NCT06740370 | Recruiting | NO | Phases not applicable | Interventional | 2024 |
| NCT06301399 | Recruiting | NO | PhaseⅡ | Interventional | 2024 |
| NCT03939975 | Completed | YES | PhaseⅡ | Interventional | 2019 |
| NCT05535998 | Completed | NO | Phases not applicable | Observational | 2022 |
| NCT06999694 | Recruiting | NO | PhaseⅡ | Interventional | 2025 |
| NCT05713994 | Recruiting | YES | Phases not applicable | Observational | 2023 |
| NCT04814030 | Unknown | NO | PhaseⅡ | Interventional | 2021 |
| NCT05937295 | Recruiting | YES | PhaseⅠ | Interventional | 2023 |
| NCT07282184 | Recruiting | NO | PhaseⅠ/Ⅱ | Interventional | 2025 |
| NCT06818097 | Completed | NO | Phases not applicable | Observational | 2025 |
| NCT07157306 | Not yet Recruiting | YES | PhaseⅡ | Interventional | 2025 |
| NCT04814043 | Unknown | NO | PhaseⅡ | Interventional | 2021 |
| NCT06632106 | Active not recruiting | NO | Phases not applicable | Observational | 2024 |
| NCT07238881 | Recruiting | NO | PhaseⅡ | Interventional | 2025 |
| NCT03966209 | Unknown | NO | PhaseⅠ | Interventional | 2019 |
| NCT06337162 | Withdrawn | NO | PhaseⅠ | Interventional | 2024 |
| NCT05809869 | Recruiting | NO | PhaseⅡ | Interventional | 2023 |
| NCT06538935 | Not yet Recruiting | NO | PhaseⅡ | Interventional | 2024 |
| NCT03869034 | Active not recruiting | YES | PhaseⅡ | Interventional | 2019 |
| NCT05913583 | Unknown | YES | Phases not applicable | Observational | 2023 |
| NCT07175441 | Recruiting | NO | PhaseⅡ | Interventional | 2025 |
| NCT05277675 | Unknown | NO | Phases not applicable | Interventional | 2022 |
| NCT06232759 | Completed | YES | PhaseⅡ | Interventional | 2024 |
| NCT05582278 | Unknown | NO | PhaseⅡ | Interventional | 2022 |
| NCT05408221 | Recruiting | NO | PhaseⅡ/Ⅲ | Interventional | 2022 |
| NCT07078305 | Not yet Recruiting | NO | PhaseⅡ | Interventional | 2025 |
| NCT06261125 | Recruiting | YES | PhaseⅡ | Interventional | 2024 |
| NCT07350824 | Not yet Recruiting | NO | Phases not applicable | Interventional | 2026 |
| NCT05233358 | Unknown | YES | Phases not applicable | Interventional | 2022 |
| NCT06632093 | Recruiting | NO | Phases not applicable | Observational | 2024 |
| NCT06423144 | Not yet Recruiting | NO | Phases not applicable | Observational | 2024 |
| NCT04652440 | Unknown | NO | PhaseⅠ/Ⅱ | Interventional | 2020 |
| NCT06029829 | Unknown | NO | PhaseⅡ | Interventional | 2023 |
| NCT07324473 | Not yet Recruiting | NO | PhaseⅡ | Interventional | 2026 |
| NCT04826406 | Unknown | NO | PhaseⅡ | Interventional | 2021 |
| NCT06370065 | Recruiting | NO | PhaseⅡ | Interventional | 2024 |
| NCT06031480 | Unknown | NO | PhaseⅡ | Interventional | 2023 |
| NCT04297202 | Unknown | YES | PhaseⅡ | Interventional | 2020 |
| NCT03859128 | Unknown | NO | PhaseⅡ/Ⅲ | Interventional | 2019 |
| NCT06333561 | Recruiting | NO | Phases not applicable | Observational | 2024 |
| NCT07075120 | Not yet Recruiting | YES | PhaseⅠ | Interventional | 2025 |
| NCT05420922 | Unknown | NO | Phases not applicable | Observational | 2022 |
| NCT06061445 | Unknown | NO | Phases not applicable | Interventional | 2023 |
| NCT04411706 | Unknown | YES | PhaseⅡ | Interventional | 2020 |
| NCT07392866 | Not yet Recruiting | NO | PhaseⅡ/Ⅲ | Interventional | 2026 |
| NCT04862949 | Completed | YES | Phases not applicable | Observational | 2021 |
| NCT03744247 | Withdrawn | NO | PhaseⅢ | Interventional | 2018 |
| NCT04696055 | Completed | YES | PhaseⅡ | Interventional | 2021 |
| NCT05582109 | Unknown | NO | PhaseⅡ | Interventional | 2022 |
| NCT06117891 | Recruiting | NO | Phases not applicable | Observational | 2023 |
| NCT03463876 | Completed | YES | PhaseⅡ | Interventional | 2018 |
| NCT05488522 | Recruiting | NO | PhaseⅠ | Interventional | 2022 |
| NCT05155189 | Recruiting | NO | PhaseⅠ | Interventional | 2021 |
| NCT03337841 | Unknown | NO | PhaseⅡ | Interventional | 2017 |
| NCT07186621 | Recruiting | NO | PhaseⅢ | Interventional | 2025 |
| NCT04913480 | Unknown | NO | PhaseⅡ | Interventional | 2021 |
| NCT03793725 | Unknown | NO | PhaseⅡ | Interventional | 2019 |
| NCT06311929 | Recruiting | NO | PhaseⅣ | Interventional | 2024 |
| NCT04947956 | Unknown | YES | Phases not applicable | Observational | 2021 |
| NCT05162352 | Completed | YES | PhaseⅡ | Interventional | 2021 |
| NCT04430452 | Recruiting | NO | PhaseⅡ | Interventional | 2020 |
| NCT04992143 | Unknown | NO | PhaseⅡ | Interventional | 2021 |
| NCT05249569 | Terminated | YES | PhaseⅡ | Interventional | 2022 |
| NCT07340502 | Not yet Recruiting | NO | Phases not applicable | Observational | 2026 |
| NCT06710223 | Recruiting | NO | PhaseⅠ | Interventional | 2024 |
| NCT04988945 | Recruiting | YES | PhaseⅡ | Interventional | 2021 |
| NCT04997850 | Completed | NO | PhaseⅠ/Ⅱ | Interventional | 2021 |
| NCT05962450 | Unknown | NO | PhaseⅡ | Interventional | 2023 |
| NCT05675462 | Unknown | NO | PhaseⅠ | Interventional | 2023 |
| NCT06741020 | Recruiting | YES | PhaseⅠ/Ⅱ | Interventional | 2024 |
| NCT06313190 | Recruiting | YES | PhaseⅡ | Interventional | 2024 |
| NCT04615143 | Recruiting | NO | PhaseⅡ | Interventional | 2020 |
| NCT07331883 | Recruiting | NO | PhaseⅡ | Interventional | 2026 |
| NCT04665609 | Unknown | NO | PhaseⅢ | Interventional | 2020 |
| NCT05178043 | Unknown | NO | PhaseⅡ | Interventional | 2022 |
| NCT06096779 | Recruiting | NO | PhaseⅡ | Interventional | 2023 |
| NCT05873244 | Recruiting | YES | PhaseⅡ | Interventional | 2023 |
| NCT05822752 | Active not recruiting | NO | PhaseⅡ | Interventional | 2023 |
| NCT06496815 | Not yet Recruiting | NO | PhaseⅣ | Interventional | 2024 |
| NCT06487559 | Recruiting | NO | PhaseⅠ | Interventional | 2024 |
| NCT05578430 | Unknown | NO | PhaseⅡ | Interventional | 2022 |
| NCT05281926 | Unknown | NO | PhaseⅠ | Interventional | 2022 |
| NCT06199297 | Completed | NO | Phases not applicable | Observational | 2024 |
| NCT04985136 | Terminated | NO | PhaseⅢ | Interventional | 2021 |
| NCT04212221 | Terminated | YES | PhaseⅠ/Ⅱ | Interventional | 2019 |
| NCT06896396 | Not yet Recruiting | NO | PhaseⅠ/Ⅱ | Interventional | 2025 |
| NCT03605706 | Unknown | NO | PhaseⅢ | Interventional | 2018 |
| NCT06041477 | Recruiting | YES | PhaseⅢ | Interventional | 2023 |
| NCT07059494 | Recruiting | NO | PhaseⅣ | Interventional | 2025 |
| NCT04183088 | Unknown | NO | PhaseⅡ | Interventional | 2019 |
| NCT06461936 | Active not recruiting | NO | Phases not applicable | Observational | 2024 |
| NCT05803928 | Recruiting | NO | PhaseⅠ | Interventional | 2023 |
| NCT07168668 | Not yet Recruiting | NO | PhaseⅡ | Interventional | 2025 |
| NCT06742424 | Recruiting | NO | PhaseⅡ | Interventional | 2024 |
| NCT06563934 | Not yet Recruiting | NO | PhaseⅡ | Interventional | 2024 |
| NCT05833126 | Unknown | NO | PhaseⅡ | Interventional | 2023 |
| ISRCTN12249513 | Completed | NO | Phases not applicable | Observational | 2025 |
| ISRCTN12669009 | Active not recruiting | NO | PhaseⅠ/Ⅱ | Interventional | 2022 |
| 2019-003337-41 | Active not recruiting | NO | PhaseⅢ | Interventional | 2019 |
| ChiCTR2600118858 | Active not recruiting | NO | PhaseⅡ | Interventional | 2026 |
| ChiCTR2500101088 | Not yet Recruiting | NO | PhaseⅠ | Interventional | 2025 |
| ChiCTR2400085146 | Not yet Recruiting | NO | PhaseⅡ | Interventional | 2024 |
| ChiCTR2400083305 | Not yet Recruiting | NO | Phase0 | Observational | 2024 |
